# Assessing the drought tolerance of some sesame genotypes using agro-morphological, physiological, and drought tolerance indices

**DOI:** 10.1186/s12870-025-06235-0

**Published:** 2025-03-18

**Authors:** KH. A. Mourad, Yasmeen Ismail Mahmoud Othman, Doha M. Kandeel, Mohamed Abdelghany

**Affiliations:** 1 Oil Crops Dept., Field Crops Res. Inst., Agric. Res. Center, Giza, Egypt; 2 Physiological Crops Dept., Field Crops Res. Inst., Agric. Res. Center, Giza, Egypt; 3 Crop Science Department, Faculty of Agriculture, Damanhour University, Damanhour, 22516 Egypt

**Keywords:** Yield stability index, Drought stress, Chlorophyll and seed yield

## Abstract

**Background:**

One significant abiotic stressor that harms sesame productivity globally is drought. This investigation used six sesame genotypes to measure variance in many variables under irrigated and terminal drought stress environments. Growth characteristics (plant height, fruiting zone length, branches’ number), yield-related parameters (capsules’ number per plant, capsule’s length, 1000 seeds’ weight, seed yield per plant, and seed yield per feddan) and physiological characters (relative water content, chlorophyll A content, chlorophyll B content, chlorophyll A + B content, and proline concentration) of sesame were measured. Six drought indices (geometric mean productivity (GMP), mean productivity (MP), stress tolerance index (STI), tolerance index (TOL), stress susceptibility index (SSI) and, yield stability index (YSI)) were derived using seed yield per feddan. This study was aimed to investigate the effects of drought stress on the physiological and yield-related characteristics of the sesame genotypes and to find the qualities that were most helpful in selecting drought-resistant genotypes.

**Results:**

The analysis of variance revealed significant differences in genotypes and water depletion ratios, as well as their interactions, for all growth variables, except the interaction between genotypes and water depletion ratios for plant height and relative water content. Line 13 (H. 102) had the highest branches’ number (6.85), capsules’ number per plant (239.33) and capsule’s length (3.35 cm) attributes under normal circumstances. Line 31 (H. 68) produced the maximum yield per plant (33.45 g) and feddan (679.83) and had the highest weight of 1000 seeds (3.9 g) under normal circumstances. Under the level (80% water depletion ratio), H. 68 had the highest amounts of chlorophyll A (5.73) and chlorophyll A + B (17.37) whereas H. 102 exhibited the highest concentration of chlorophyll B (5.73). The genotype H. 68 of sesame was found to have the greatest MP (650.35), GMP (649.32) and YI (1.16) indices followed by genotype H. 102. The Shandaweell 3 genotype resulted in the lowest SSI (36.92) and TOL (0.55) indices. Line 26 (H132) exhibited the highest average YSI values.

**Conclusions:**

These data revealed that genotypes H. 102, H. 68 and Shandaweell 3 are the most drought-tolerant among the genotypes utilized in this study. These results may contribute to developing effective breeding techniques for drought-stressed sesame in the future.

## Introduction

Drought, heat and salinity are the main abiotic variables that reduce sesame productivity and output [1]. High degrees of drought stress have a detrimental effect on sesame productivity and quality, despite its relative tolerance to water scarcity if compared to other oilseed crops [2]. Over the world, drought is a major factor in crop losses, with average yields decreasing by more than 50% [3]. Researching a range of drought-related traits is essential to efficiently select genetic material since the intricate trait of drought tolerance in crops is controlled by multiple processes that commonly cooperate [4].

Water makes up between 80 and 95% of the fresh biomass of the plant body and is essential to many physiological processes, including many facets of plant growth and metabolism [5]. Drought stress negatively impacts photosynthetic activity, agricultural yield, and crop quality by lowering the pressure of turgor, exchange of gases, and water content in crops [6]. Water deficiency causes oxidative stress by producing partially reduced forms of atmospheric oxygen, including superoxide radical [O_2_^−^], hydroxyl radical [HO^−^], hydrogen peroxide [H_2_O_2_], and oxygen singlet [O_2_] [7]. Global population growth and climate change are exacerbating the issue of drought stress, which has a substantial impact on grain output and quality worldwide [8]. Prolonged dehydration can stop photosynthesis and cause metabolic problems. Genotypes with different levels of drought resistance are crucial for crop breeding during dry spells [9]. In addition to how the plants interact with the soil and atmosphere, plant responses to drought stress might differ based on the timing, intensity, length, and frequency of the stress [10]. Selecting cultivars resistant to drought often involves primary selection criteria, such as grain yield, and secondary characteristics associated with drought [11]. Producing high-yielding cultivars during drought conditions is a major problem for breeders [12]. A potential solution to these issues is to choose new drought-tolerant varieties and generate large amounts of grain and oil [13].

A common component in cuisines worldwide is sesame [*Sesamum indicum* L], an ancient oil crop. Sesame is a diploid plant [2n = 26], growing as a shrub [14]. It is among the oldest crops farmed in Africa and Asia [15]. Sudan is the world’s leading producer of sesame seed, accounting for 22.42% of global production. In 2023, the top three nations own a share of 41.3%, while the ten largest countries possess approximately 76.4% [16]. Among the healthiest oilseeds, sesame has seeds that are high in oil [50–62%], proteins [18–25%], carbs [13–25%], and easily digested fibers [9–11%] [17]. Furthermore, sesame seeds have a high concentration of vitamins, minerals, and lignans, which have a variety of nutritional, pharmaceutical, and industrial applications [18]. Due to its high percentage of unsaturated fatty acids [~ 85%] and antioxidants [including sesamin, sesamolin, and tocopherols], sesame oil is said to be of outstanding quality. Sesamin and sesamolin, the two primary lignans found in sesame, offer several health benefits, including anti-inflammatory, antioxidant, hypocholesterolemic, and antihypertensive effects [19]. Regarded as both a spice and an oil seed, sesame seeds provide a balanced source of oil [20]. Compared to most other popular oil seeds, it has roughly 50% more oil [20]. One of the priciest and most sought-after edible oils in the world is sesame seed oil. It is among the healthiest oils since it contains high levels of unsaturated fatty acids, such as 38.84% oleic acid and 46.26% linoleic acid, validating sesame seed oil’s placement of its oils in the group of oleic-linoleic acid-carrying oils [21]. Furthermore, the oxidative stress and lipid profile might be greatly impacted by sesame seed oil’s greater phytoestrogen and lignin contents [20]. Although sesame is a long-standing and significant edible oilseed crop, it has received less attention and use than other crop species; as a result, there is still tremendous potential for genetic improvement [22].

One of Egypt’s most significant oil crops, sesame boosts the country’s economy, raises national income, and lowers imports [23]. Expanding oil crop cultivation in recently recovered lands can assist in meeting the country’s growing demand for oil sources and local production shortages [24]. Sesame is one of the best crops to grow in the newly reclaimed lands, which are being expanded on a large scale outside Egypt’s Nile Valley due to it is short-lived [3–4 months], requires minimal water, and is resistant to drought [24]. Sesame is a vital oilseed crop in Egypt, as most seeds are ingested directly. Sesame production in Egypt reached approximately 578 tons per feddan in 2021, with an area under cultivation of 90.39 thousand feddans [25]. Water security and the lack of edible oil are two of Egypt’s biggest issues [26]. Egypt’s output and consumption of sesame are estimated at 38% apart [27]. The sesame food gap can be reduced by cultivating high-yielding varieties under drought stress.

Crop drought tolerance is determined by analyzing morphological, physiological, anatomical, and molecular traits [28, 29]. The combination of morphological, agronomic, and physiological features may make it easier to identify genotypes resistant to drought [30]. Numerous physiological characteristics, such as relative water content—which is maintained higher in tolerant plants—and proline buildup play a major role in determining the relationship between plants and water [31]. Additionally, many indicators based on seed yield have been put forth to assess and choose genotypes that can withstand drought under stressful circumstances [32]. Among these metrics are the geometric mean productivity, yield index, stress tolerance, stress sensitivity, mean productivity and relative drouth index [33]. This research aimed to examine how six sesame genotypes’ yield-related and physiological parameters were affected by drought stress and to determine which traits were most useful for identifying genotypes that were resistant to drought.

## Materials and methods

### Plant materials

New Six promising Egyptian sesame lines obtained from the Field Crop Research Department, Agriculture Research Centre, Egypt, were used in this investigation, including lines 6, 13, 16, 26, 31 and Shandaweell 3 (Table 1).

**Table 1:** List of genotypes used

No.	Genotypes	Origin and pedigree
6	N. A259	U.S.A
13	H. 102 Family _28−1_	Egypt
16	H.106 Family _13_	Egypt, a line selected from N.A114 x N.A247
26	H132 Family _2_	Egypt
31	H. 68 Family _21_	Egypt, a line selected from line B21 x line 574
--	Shandaweel 3	Egypt 1987, a line selected from Giza32 x N. A. 130

### Field experiments and studied traits

The field experiment was carried out over two seasons, 2022 and 2023, in the experimental farm of Itay El-Baroud Research Station in El-Beheira Governorate, Egypt. This investigation examined three water irrigation treatments, including normal irrigations (40% depletion water ratio), Water Deficit 1 (60% depletion water) and Water Deficit 2 (80% depletion water ratio). The irrigation treatments were assigned to the main plots while sesame genotypes were randomly distributed in the subplots in a split-plot design with three replications. Irrigation scheduling was made at three levels of soil moisture depletion (40, 60 and 80%) using the CROPWAT model version 8.0 after feeding it with crop, soil, and weather data [34]. Each sesame farming technique followed the Ministry of Agriculture and Land Reclamation’s recommendations.

### The examined parameters

#### The agro-morphological characteristics

At the end of the drought stress phase, ten plants were randomly selected from the outer ridges of each experimental unit, and the following parameters were noted; plant height (PH, cm), seed yield per plant (YP, g), seed yield per feddan (YF, kg), fruiting zone length (cm), number of branches, number of capsules per plant, the length of the capsule (cm), and the weight of 1000 seeds (g). After laying a polyethylene sheet over the ground beneath the plants, the inner three ridges were used to calculate the seed yield (kg/fed). Following the collection of the seeds as swathed upright bundles, drying, conveying, and translating operations to thresh and clean, the weight of the adjusted seeds was collected immediately after, and the seed yield was then estimated.

### Physiological characters

The physiological measures were taken on the third completely formed leaf from the plant’s apex.

During the morning (about 8:00 am), the most developed leaf tissues excised were used to calculate the percentage of relative water content (RWC). The fresh weight (FW) of the removed leaves was determined, and they were subsequently rehydrated at room temperature in a Petri plate filled with water. To calculate DW, turgid weight (TW) was calculated by allowing leaves to fully rehydrate (16 h), removing all water from their surface, weighing the leaves, and then drying them at 70 °C for 48 h.

The following equation was used to determine the relative water content:

RWC (%) = [(W-DW) / (TW-DW)] x 100,

Where,

W – Sample fresh weight.

TW – Sample turgid weight.

DW – Sample dry weight [35].

Following Fernandez [36] Methodology, the levels of chlorophyll A, B, and total chlorophyll were extracted and calculated as follows: A known volume of N-dimethylformamide (6 ml) was used to extract the chlorophyll content from two discs (0.8 mm) of the most developed leaf. Using spectrophotometry, the absorbance of the extracted color was determined at (664) and (647) nm.

The following formula was used to determine the amounts of chlorophyll A and B:

Chlorophyll *a* = 12.64 X A664–2.99 X A647 = mg/l.

Chlorophyll *b* = 23.26 X A647–5.6 X A664 = mg/l.

Chlorophyll *a* + *b* = 7.04 X A664 + 20.27 X A664 = mg/l.

Where: A (664) is the reading at 664 nm.

A (647) is the reading at 647 nm.

The concentration of chlorophyll contents was then expressed as mg dm-2.

The method outlined by Rosielle and Hamblin [37] was used to determine the proline content.

### Drought indices

Under both normal and drought conditions, the following formulas were used to calculate the seed yield fed-1 under each condition: geometric mean productivity (GMP), mean productivity (MP), tolerance index (TOL), stress susceptibility index (SSI), yield stability index (YSI), drought resistance index (DI), relative drouth index (RDI) and yield index (YI).

1. GMP=√Ys×Yp [38].
2. MP = (Yp + Ys) /2 [39].
3. TOL = Ys-Yp [37].
4. SSI=(1-(Ys / Yp)) / (1-(Ӯs / Ӯ p)) [40].
5. YSI = Ys/Yp [41].
6. DI= [𝑌𝑠𝑥(𝑌𝑠/𝑌𝑝)]/𝑌̅s [42].
7. RDI= (𝑌𝑎𝑐𝑡.𝑖 − 𝑌𝑒𝑠𝑡.𝑖)/SE of − 𝑌𝑒𝑠𝑡.𝑖) [43].
8. YI= 𝑌𝑠/ 𝑌̅s [44].

Ys represents the genotype yield under stress, and Yp represents the genotype yield under normal circumstances. The aggregate means of all lines under normal and drought conditions are Ӯp and Ӯs. 𝟏 − 𝒀𝒔/𝒀𝑷 is the stress intensity. The values of 𝒀𝒂𝒄t.𝒊, 𝒀𝒆𝒔t.𝒊, and (S. E. of 𝒀𝒆𝒔𝒕) correspond to the actual yield under stress, the predicted yield determined using regression under stress, and the standard error of the estimated grain yield across all genotypes, respectively.

### Statistical analysis

SAS v9.1 was used to conduct variance and ANOVA analyses in a combined split-plot design of the examined data. A homogeneity test was performed across all attributes. R v4.3.2 was used to calculate Pearson’s correlation coefficients between the various parameters. To compare the treatment means, the least significant differences (LSD) at the 0.05 probability level were used.

## Results

### Agro-morphological characters

#### Growth parameters

Under both normal and water-stressed conditions, the plant’s height, fruiting zone length, and number of branches were measured. The analysis of variance found substantial differences in genotypes and depletion ratio of water and their interactions for all growth variables except for the interaction between genotypes and depletion ratio of water for plant height trait.

**Fig. 1:**
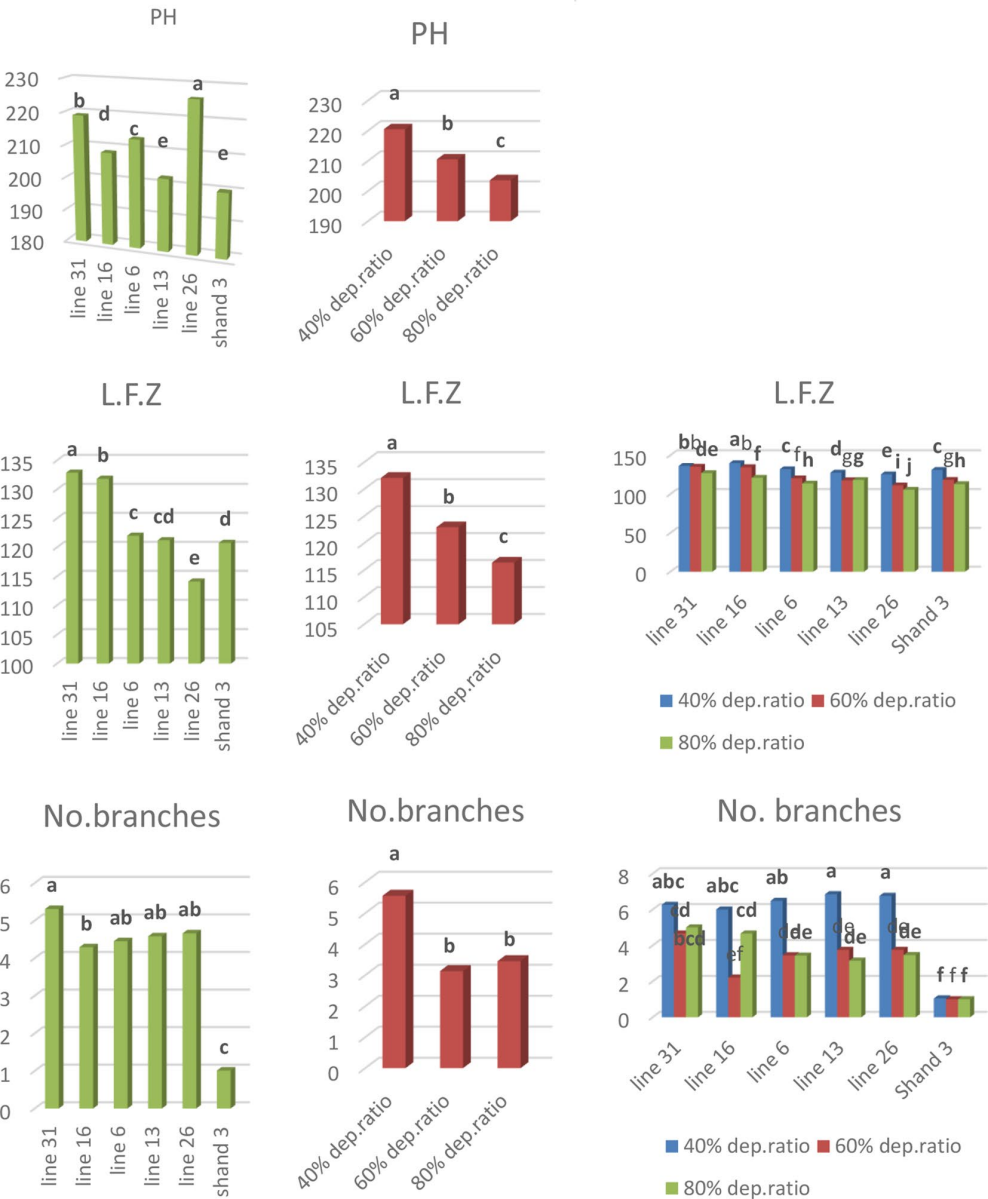
Impact of irrigation water treatments, genotype and their interactions on plant height, fruiting zone length, and number of branches characteristics

At 225.97 cm, H132 had the tallest plant due to the genotype impact. The shortest genotype, nevertheless, was Shandaweel 3 (199.97 cm). Line 26 had the smallest fruiting zone, measuring 114.11 cm, while H. 68 had the greatest, measuring 132.88 cm. According to Fig. 1, Shandaweel 3 had the fewest branches (1.01) and H. 68 had the most (5.31) (Fig. 1).

All development traits responded differently depending on the degree of water stress. Among all genotypes, the maximum average plant height (220.48 cm), fruiting zone length (132.08 cm), and branch count (5.56 cm) were produced under normal conditions (40% depletion water ratio). However, the water-stressed stage (80% depletion water ratio) had the lowest plant height (203.59 cm), fruiting zone length (116.38 cm), and branch count (3.45) (Fig. 1).

The interactions of water-stressed levels and genotypes revealed that under normal conditions, H. 106 produced the longest fruiting zone length (139.83 cm). In contrast, H132 in the water-stressed stage (80% depletion water ratio) produced the shortest fruiting zone length (105.66 cm). In terms of the number of branches, H. 102 under normal conditions produced the most (6.85), whereas line Shandaweel 3 produced the fewest under both water-stressed stages (60% and 80% water depletion ratio) (Fig. 1).

**Fig. 2:**
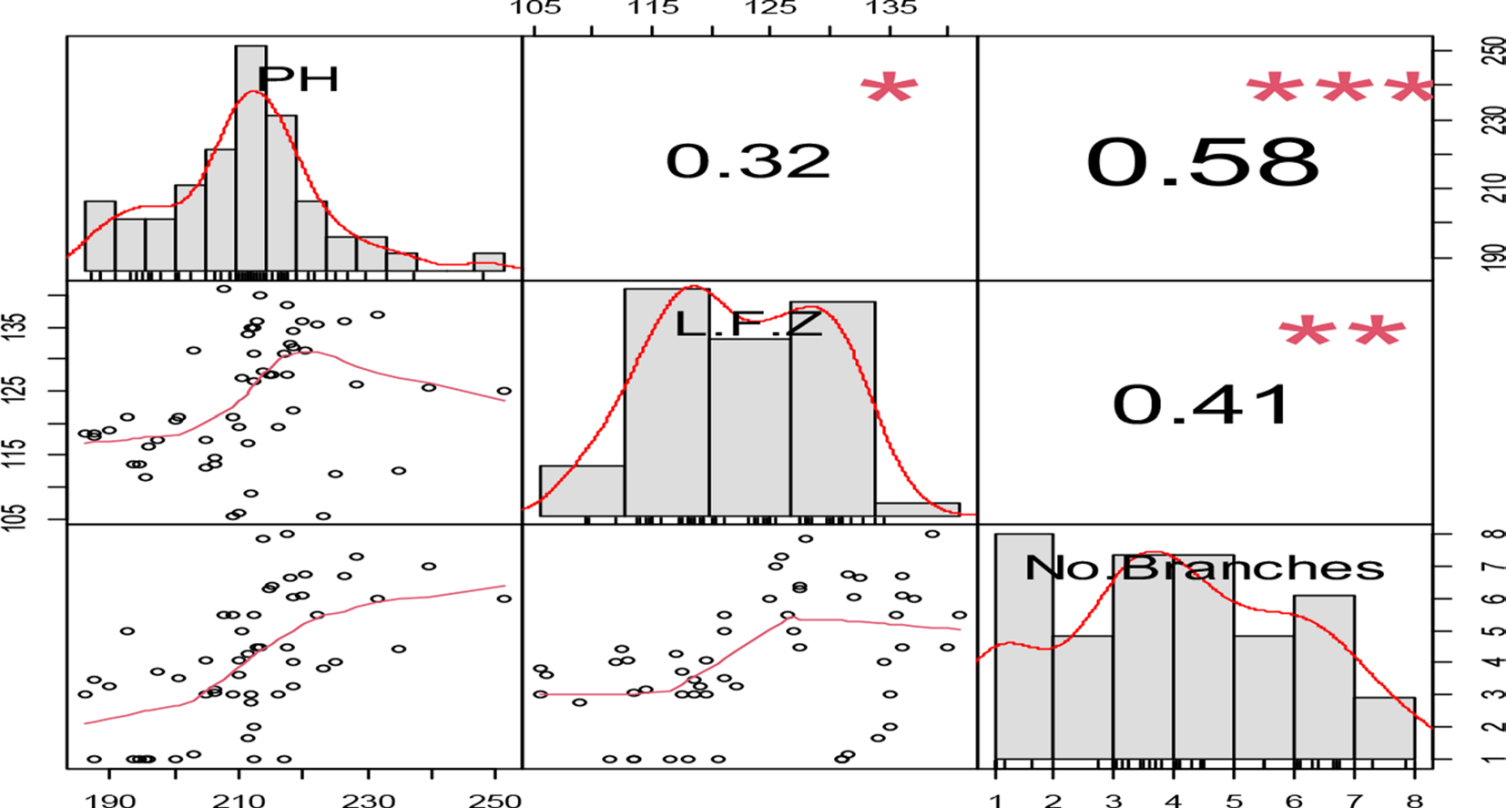
The associations between plant height, fruiting capsule length, and number of branches with water level depletion ratios and genotypes. The numbers on the graph represent the correlation coefficients for the three attributes. The diagonal represents the frequency distribution for each of the three qualities. The scatter distribution of the three qualities is shown from left to bottom

All growth parameters were significantly associated (*P* < 0.05). The highest association was found between plant height and the number of branches (*r* = 0.58), while the lowest correlation was found between plant height and the length of the fruiting zone features (*r* = 0.32) (Fig. 2).

### Yield-related parameters

The number of capsules per plant, the length of the capsule, the weight of 1000 seeds, the seed yield per plant, and the seed yield per feddan were assessed under normal and water-stressed conditions. The analysis of variance revealed highly significant variations in genotypes and the depletion ratio of water and their interactions for all yield variables between drought stress and non-stress environments.

**Fig. 3:**
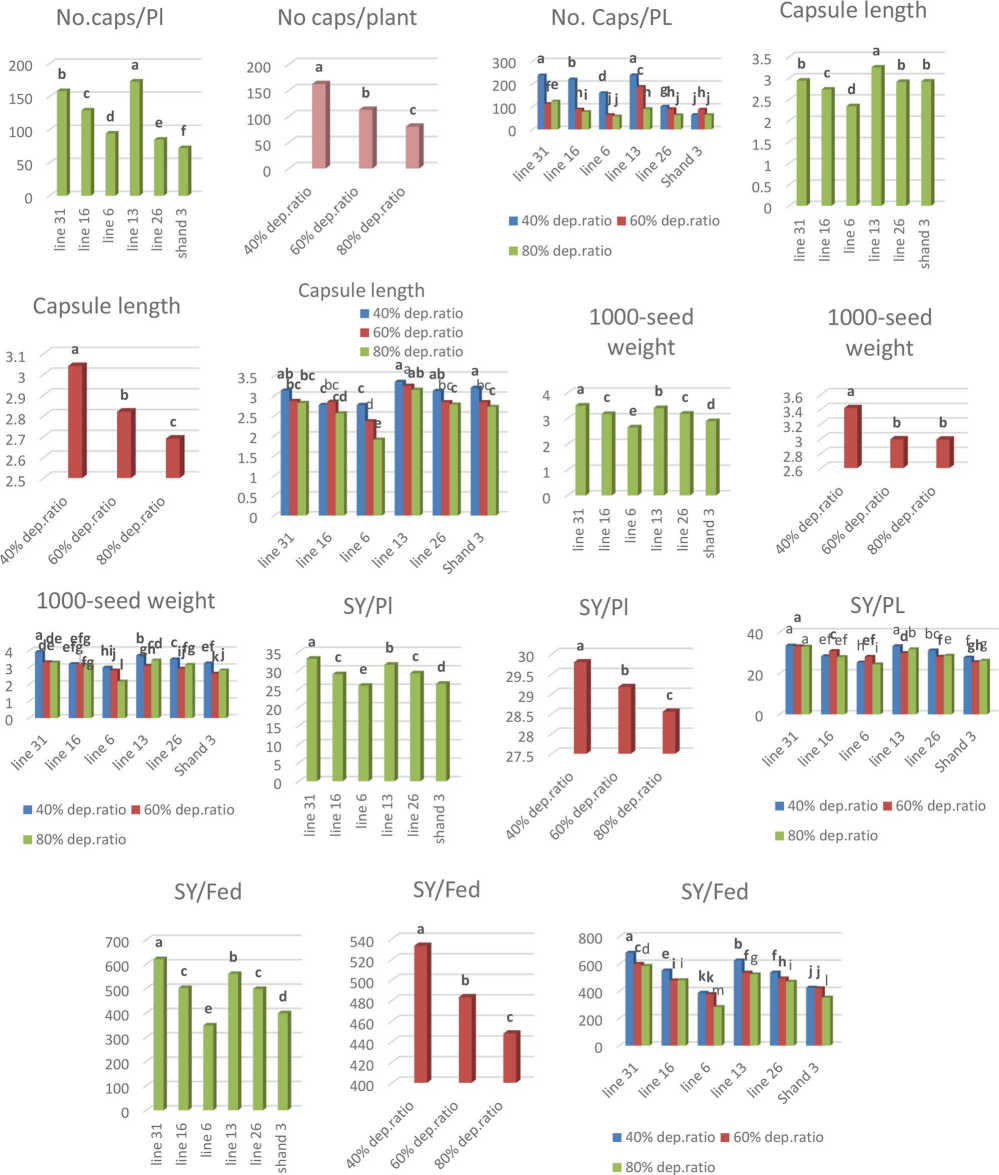
Genotype, water irrigation, and interaction effects on the number of capsules per plant, capsule length, 1000-seed weight, number of seeds generated per plant, and feddan characteristics

H. 102 had the most capsules per plant (172.66) when the genotype effect was taken into account. However, the Shand 3 genotype had the lowest score (72.05). H. 102 ‘s capsule was the longest (3.25 cm), while N. A259’s was the smallest (2.34 cm). The lowest and highest thousand seed weights, 2.65 g and 3.49 g, respectively, were found on genotypes N. A259 and H. 68. H. 68 had the highest output (33.15) in terms of the number of seeds produced per plant, whereas N. A259 had the lowest yield (25.82) (Fig. 3). N. A259 had the lowest seed output per feddan (348.69 kg), whereas the H. 68 genotype had the highest yield (620.88 kg).

Levels of water stress-induced variations in the responses of all yield-related characteristics. All genotypes generated the greatest number of capsules per plant (162.30), length of capsules (3.04 cm), the thousand seed weights (3.41 g), seed yield per plant (29.79 g), and seed yield per feddan (533.29 kg) under the normal conditions (40% depletion water ratio) (Fig. 3). However, during the water-stressed stage (80% depletion water ratio), the number of capsules per plant (80.47), length of capsules (2.69 cm), the thousand seed weights (2.98 g), seed yield per plant (28.55 g), and seed yield per feddan (447.94 kg) were all lowest.

In terms of the number of capsules per plant, the interplay between genotypes and water-stressed levels showed that H. 102 had the most capsules (239.33) under normal conditions, followed by H. 68 (238.66) under the same conditions (Fig. 3). On the other hand, under water-stressed conditions (80% depletion water ratio), N. A259 and H132 had the fewest capsules (58 and 63.5, respectively) (Fig. 3). H. 102 in non-stressed conditions (40% depletion water ratio) had the longest capsule (3.35 cm). N. A259 under water-stressed phases (80% depletion water ratio) had the smallest capsule length (1.9 cm) (Fig. 3).

H. 68 had the largest weight of 1000 seeds under normal conditions (3.9 g), whereas N. A259 had the lowest weight (2.16 g) under water-stressed stages (80% depletion water ratio) (Fig. 3). The best yield per plant and feddan was obtained during the normal conditions on H. 68, which recorded 33.45 g and 679.83 kg respectively. In contrast, the lowest yield per plant and feddan was obtained under water-stressed stages (80% depletion water ratio) on N. A259, which recorded 24.30 g and 282.6 kg respectively (Fig. 3).

**Fig. 4:**
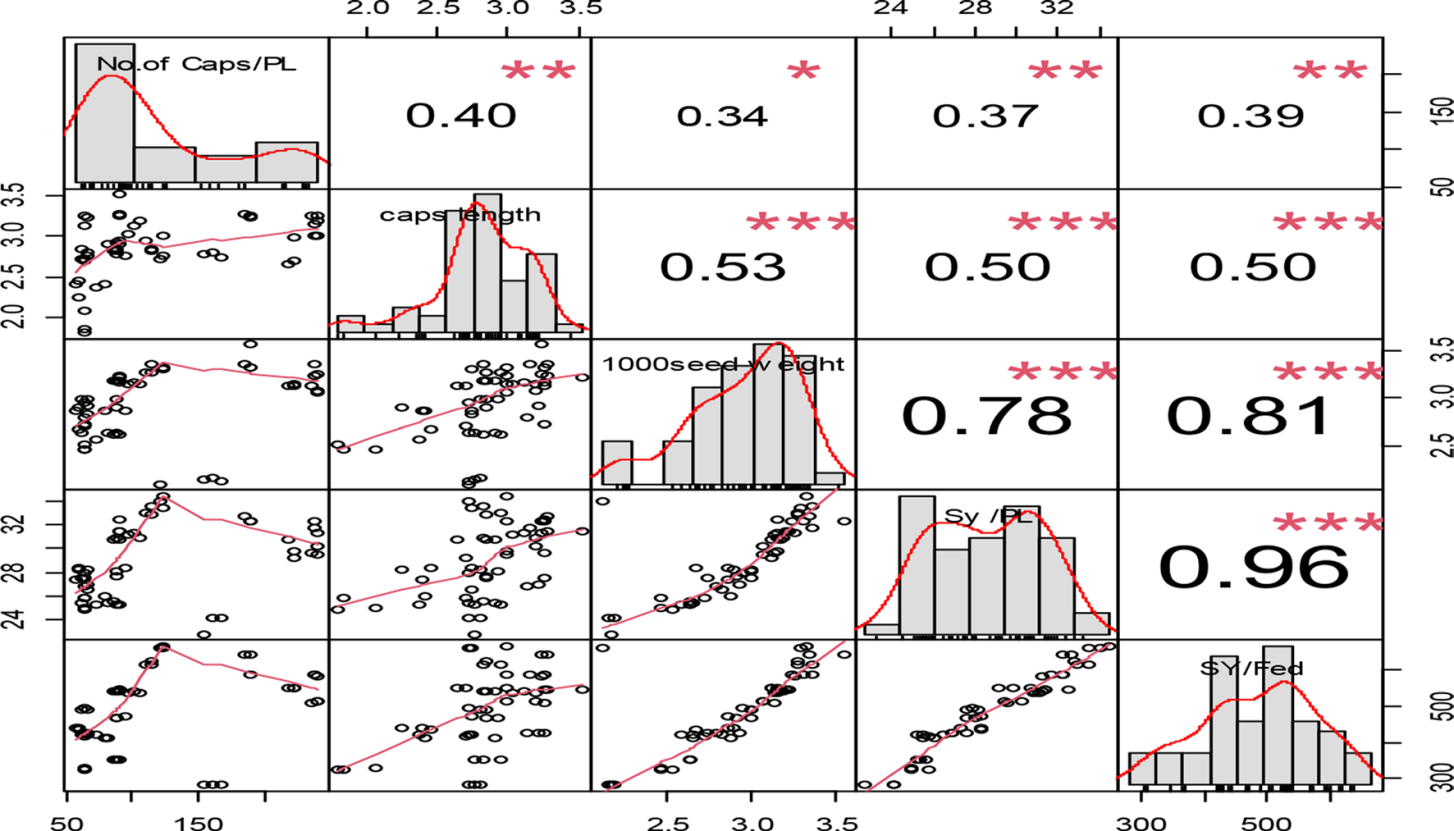
The correlations between the number of capsules per plant, capsule length, 1000-seed weight, seed yield per plant, and seed yield per feddan over water level depletion ratios and genotypes. The right-top depicts the correlation coefficients among the five attributes. The diagonal represents the frequency distribution for each of the five attributes. The left-bottom displays the scatter distribution among the five attributes

A substantial association (*P* < 0.001) was discovered between all yield-related characteristics (Fig. 4). The most significant correlation (*r* = 0.96) was discovered between seed yield per plant and seed yield per feddan (*P* < 0.001). The lowest association (*r* = 0.34) was found between the number of capsules per plant and the 1000-seed weight characteristics (Fig. 4).

### Physiological characters

Normal and water-stressed conditions were used to determine relative water content, chlorophyll *a* content, chlorophyll *b* content, chlorophyll *a* + *b* content, and proline concentration. In terms of water stress levels and genotype effects, the analysis of variance revealed that water stress levels and genotype effects had significant impacts on all variables except chlorophyll *a* content, chlorophyll *a* + *b* content and proline content traits, where the genotype effects were inconsequential. The water depression ratio had no significant effect on the proline content characteristic. All traits exhibited substantial effects from the genotype x water stress level interaction except relative water content.

**Fig. 5:**
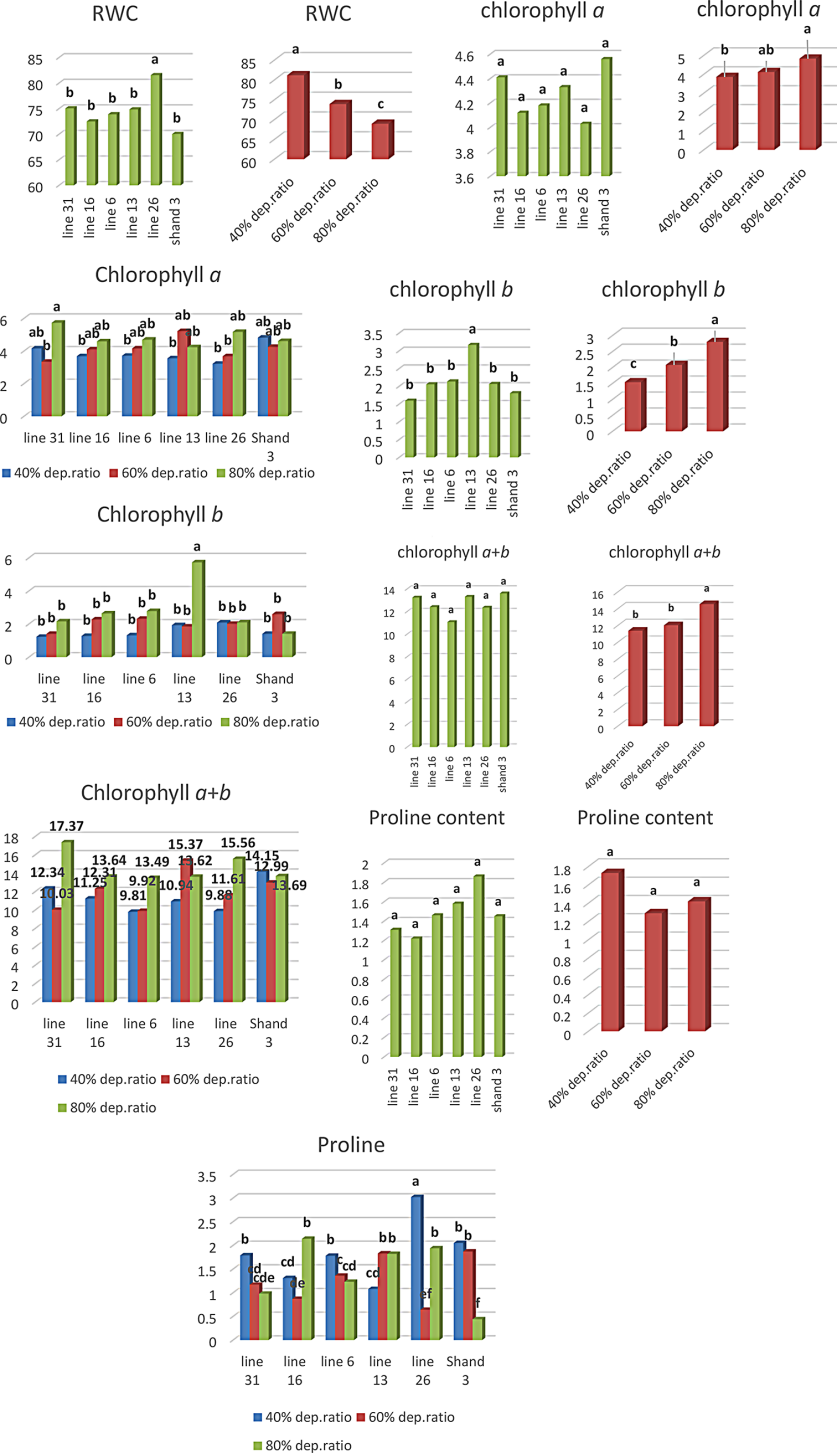
Influence of genotype and irrigation water treatments, as well as their interactions, on the properties of relative water content, proline content, chlorophyll *a*, chlorophyll *b*, and chlorophyll *a* + *b*

Across all water stress levels, the relative water and chlorophyll *b* content characteristics exhibited distinct behaviors in response to genotype treatment. Shandweel 3 had the lowest relative water content (70.06) and H132 had the highest (81.56). Regardless of the degree of water stress, H. 102 had the highest concentration of chlorophyll B (3.17), whereas H. 68 had the lowest (1.60) (Fig. 5).

Every physiological measure, except for proline content, reacted differentially to varying degrees of water stress throughout the genotype treatments. The highest level of water stress (81.16) across all genotypes was associated with the relative water content characteristic (40% water depletion ratio). On the other hand, the water stress level (80% water depletion ratio) was the lowest (68.94) (Fig. 5). The properties of chlorophyll *a*, chlorophyll *b*, and chlorophyll A + B were most affected by the water stress level (80% water depletion ratio), with respective values of 4.83, 2.80, and 14.56. Conversely, the usual conditions (40% water depletion ratio) had the lowest values, with respective readings of 3.86, 1.54, and 11.39 (Fig. 5).

Water-stressed levels and genotype treatments interaction revealed that the maximum chlorophyll *a* (5.73) was produced for genotype H. 68 under water-stressed levels (80% water depletion ratio). By comparison, H132 produced the least amount of chlorophyll *a* (3.22) under normal conditions. H. 68 had the lowest chlorophyll *b* (1.23) under normal circumstances, but H. 102 had the most chlorophyll B under water-stressed conditions (80% water depletion ratio) (5.73). When water was stressed (80% water depletion ratio), H. 68 had the greatest chlorophyll *a* + *b* (17.37), while N. A259 had the lowest chlorophyll *a* + *b* (9.81) when water was not stressed (40% water depletion ratio). For proline content, in normal conditions, H132 had the highest proline concentration (3.02), but in water-stressed conditions (80% water depletion ratio), Shandweel 3 had the lowest proline level (0.44) (Fig. 5).

**Fig. 6:**
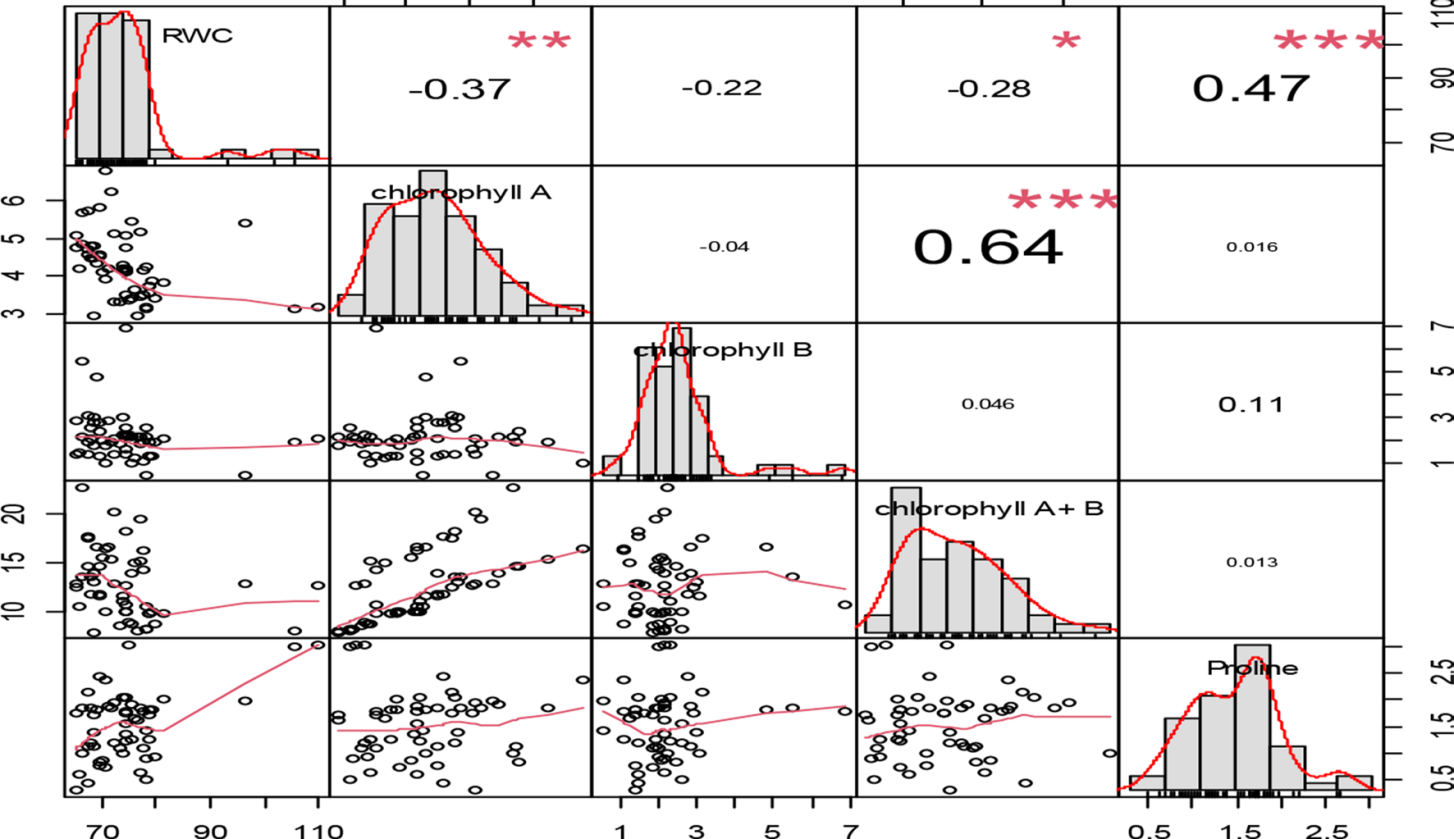
The associations between relative water content, chlorophyll *a* content, chlorophyll *b* content, chlorophyll *a* + *b* content as well as proline content over water level depletion ratios and genotypes. The numbers show the correlation coefficients among the five characteristics. The frequency distribution for each of the five qualities is shown on the diagonal. The scatter distribution between the five qualities is shown on the left bottom

The relationship between relative water content and both chlorophyll A content, chlorophyll *a* + *b* and proline content characteristics were significantly correlated. There was also a highly significant correlation between chlorophyll A content and chlorophyll A + B content. There was a negative association found between the relative water content characteristic and the levels of both chlorophyll *a*, chlorophyll *b* and chlorophyll *a* + *b* (Fig. 6). Furthermore, the strongest association (*r* = 0.64) was seen between the chlorophyll *a* content characteristic and chlorophyll A + B content followed by the association between relative water content and proline content (*r* = 0.47).

### Drought tolerance indices

**Fig. 7:**
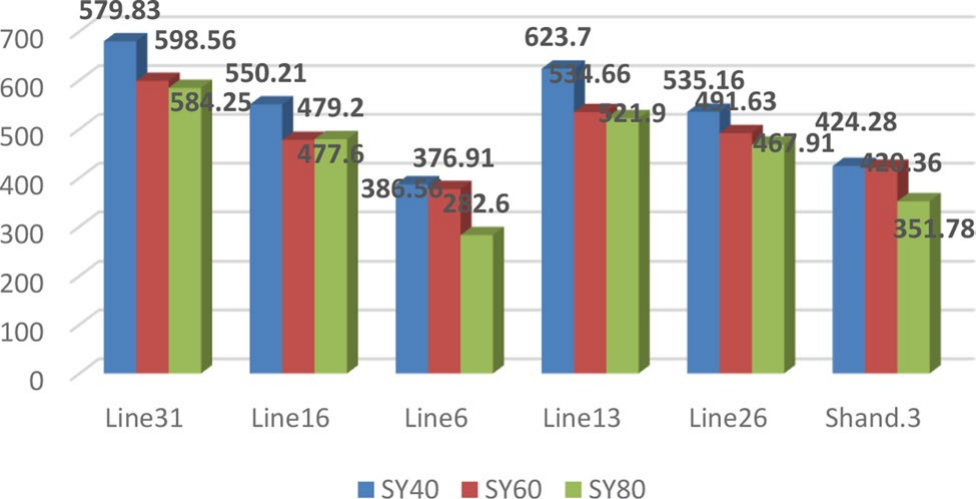
The mean amount of seed yield (kg/Fed) for the WW and WS conditions

A significant difference was found between the sesame genotypes under the varied water levels when the Ys, Yp, and drought tolerance indices were analyzed, suggesting a high level of genetic variability. Based on seed yield (kg/Fed) under WW (Yp) and WS (Ys) conditions, drought tolerance indices were computed. Under WW conditions, the average seed yield varied from 424.28 to 679.83 kg/Fed. Under moderate WS conditions, it varied between 376.91 and 598.56 kg/Fed. For severe drought stress, it varied between 282.6 and 584.25 kg/Fed (Fig. 7).

**Fig. 8:**
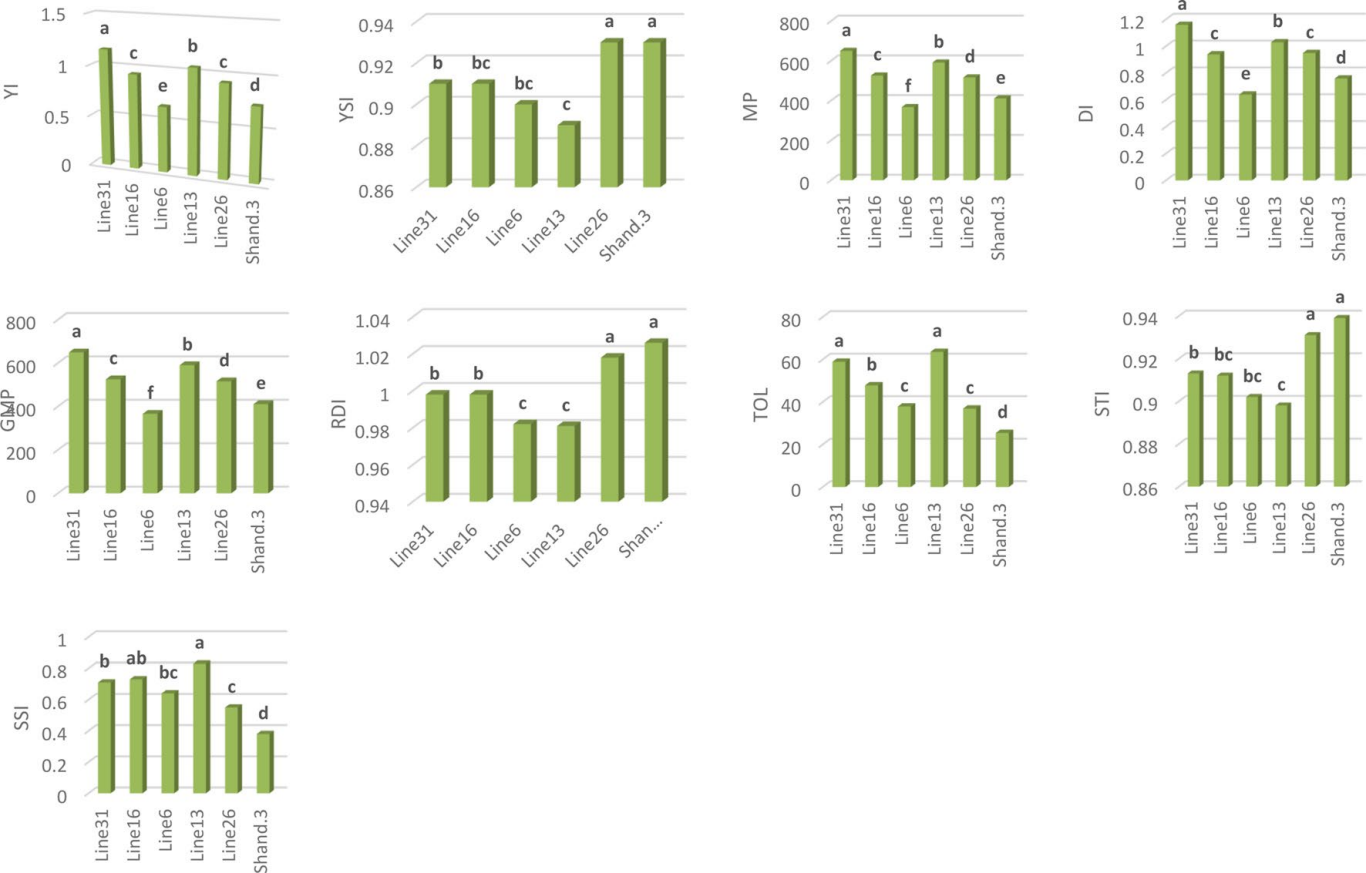
Genotypes’ effects on the drought tolerance indices (YI, YSI, MP, GMP, DI, RDI, TOL, STI and SSI)

With SSI and TOL of 0.83 and 63.61, respectively, sesame genotype H. 102 had by far the highest values. In contrast, Shandweel 3’s genotype exhibited the lowest TOL and SSI, measuring 0.55 and 36.92, respectively (Fig. 8).

The sesame genotype H. 68 recorded the highest MP and GMP, at 650.35 and 649.32, respectively. The H. 102 variety came in second, with MP and GMP recorded at 591.89 and 590.48, respectively. In contrast, the genotype “N. A259” records 367.63 and 366.26 MP and GMP, respectively, the lowest values (Fig. 8).

H132 had the lowest mean YI value, reported at 0.93, while H. 68 had the highest, recorded at 1.16. The mean YSI value of genotype H132 was the highest at 0.93, while the mean YSI value of H. 102 was the lowest at 0.89 (Fig. 8). The genotype “Shandweel 3” had the highest STI, measuring 0.939. On the other hand, the genotype “H. 102” had the lowest score, 0.898 (Fig. 8). With a DI of 1.16, genotype “H. 68” got the highest DI. However, with a score of 0.64, the genotype “N. A259” scored the lowest. With an RDI of 1.026, the genotype “Shandweel 3” had the highest. Conversely, the genotype designated as “H. 102” exhibited the lowest score of 0.981 (Fig. 8).

**Fig. 9:**
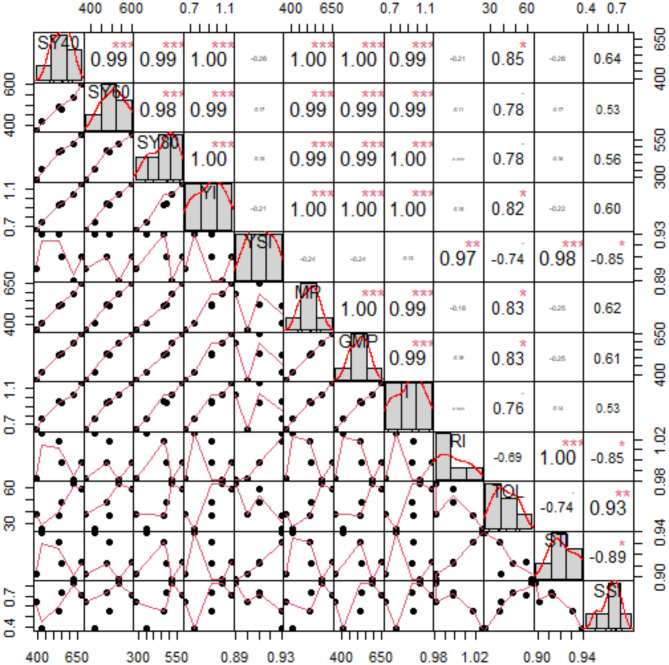
The relationships between sesame line drought indices and Yp, Ys. The characteristics’ correlation coefficients are displayed numerically. Each of the characteristics’ frequency distributions is displayed on the diagonal. The scatter distribution of the attributes is displayed at the bottom

Significant and favorable correlations were discovered between Yp and MP (1.00***), GMP (1.00***), YI (1.00***), DI (0.99***), and TOL (0.85*). Positive relationships were detected between Ys 60 (moderate drought) and YI (0.99***), MP (0.99***), GMP (0.99***), and DI (0.99***). YS 80 (extreme drought) and YI (1.00***), DI (1.00***), MP (0.99***), and GMP (0.99***) showed a very strong association (Fig. 9).

## Discussion

### Effect of drought on growth parameters of sesame

Crop plant drought tolerance comprises complex and diverse physiological and molecular mechanisms that are still poorly understood [45]. According to Yemata and Bekele [46], one of the major factors restricting crop yield worldwide is drought. Although sesame is mostly grown in dry and semiarid regions, little research has been done to determine whether sesame is drought-tolerant. Therefore, it is crucial to choose superior lines when there is water scarcity to ensure sustained sesame production [47]. Even though Golestani and Pakniyat [33] state that sesame is a drought-tolerant crop, water scarcity inhibits plant growth and blossom production, which in turn lowers the creation of capsules and seeds [45].

The current study’s findings demonstrated that variations in water levels significantly impacted the various agronomic traits. The findings showed that, in contrast to physiological processes, water stress had a notable impact on growth indices, particularly plant height and the number of branches per plant [48]. Bhargava and Sawant [9] investigated how water stress impacts sesame growth and discovered that a reduction in cell division, elongation, and differentiation results in a decrease in leaf area, which is correlated with water availability. These findings suggest that a decrease in leaf expansion may be linked to a decrease in growth. More than any other aspect, genetic features have the greatest influence on plant height traits [49]. Many Other investigations have also demonstrated plant height reduction under water stress [50, 51]. Drought has also been shown to have negative impacts on the height of plants for a variety of crops, including sugarcane [52], rice [53], and maize [51]. Reduced cell growth [54] and compromised mitosis [55] during drought environments may be the cause of a reduction in plant height. Insufficient soil moisture brought on by a lack of water can also restrict the uptake of nutrients resulting in a shorter length of plant [56]. Under WS conditions, all genotypes showed a total suppression of branch number per plant and this aligns with the findings of other investigations, including [49, 57, 58]. Lack of water during crucial growth stages, including the vegetative stage, lowers the energy available for the initiation and development of lateral buds, resulting in fewer branches. Sesame plants under drought stress typically generate fewer branches than well-watered controls [59].

### Impact of drought on sesame yield-related characteristics

It is crucial to maintain the production potential of sesame genotypes under WS conditions. Also, it is crucial to assess how sesame genotypes’ yield-attributing features relate to yield under WS. Drought has an impact on yield [15], capsule growth and development, and sesame production [57]. Drought in sesame has been demonstrated to impact yield-attributing characteristics and reduce production by 28% adversely [51]. According to our research findings, different genotypes of sesame exhibit varying reactions to water stress for yield-related traits [60]. also discovered substantial differences in the number of capsules and grain yield between sesame varieties. These Drought-adaptive genotypes are valuable resources for studying crop plant adaptation [59]. According to our findings also, yield-related attributes were lower under WS than under WW due to drought. It might be because the sesame crop is more vulnerable to drought stress during the reproductive phase than the vegetative phase, and water scarcity during the reproductive period lowers seed yield plants [13]. The decrease of sesame production and yield components under drought stress conditions may be also because of decreasing food absorption from soil osmotic loss and reduced plant water content [61].

Drought has a more detrimental effect on seed output than other yield-related traits. Sesame seed output was found to be significantly reduced by moderate drought stress. Compared to plants under drought stress at the vegetative stage, those under water deficiency during the flowering and pod-filling stages showed greater output losses [58]. Jahan et al. [62] claim that sesame production declines due to fewer seeds being produced during water-stress conditions. Additionally, under water stress, there was a reduction in capsule length, which is consistent with [50] findings as they found that the capsule length decreased from 22.7 to 14.8 mm during water stress. According to [63], drought stress during the blooming stage significantly affects sesame production characteristics such as capsule length, seed quantity, and 1000-seed weight. Goodman’s [64] study found that extreme drought stress (irrigation up to 50% flowering) reduced sesame yield, including capsule number, seed number, and 1000-seed weight, as compared to non-stressed conditions.

### Effects of drought on the physiological properties of sesame

Characters associated with drought tolerance, such as yield components and physiological traits, are efficient indicators for choosing drought-tolerant genotypes in breeding programs to reduce the impact of water scarcity on crop production [63]. Based on particular physiological features, efforts have been undertaken to improve the effectiveness of selection for drought-tolerant genotypes [65]. By identifying the physiological mechanisms that enable plants to adapt to water scarcity and maintain growth and productivity in stressful situations, tolerant genotypes may be more easily identified and used in breeding programs [66]. Thus, yield-based selection methods would be significantly enhanced by using physiological parameters as an indirect selection strategy.

Leaf RWC is regarded as one of the most significant physiological markers of stress intensity [67]. The sustained RWC under WS circumstances shows that osmotic adjustment can sustain physiological processes and cell turgescence [68]. Plant-water relations are significantly influenced by the relative water content of the plant. Drought-stressed crops exhibit a discernible decrease in the relative water content (RWC) of their leaves when compared to crops that get regular irrigation [65]. We found that RWC decreased by around 15% in the current study under WS conditions; comparable results have been published by [69]. Agronomic techniques including irrigation timing, and choosing drought-tolerant sesame types can improve the maintenance of RWC under drought stress [70]. The higher chlorophyll content is found in plants growing under WS [71], while lines resistant to drought have higher chlorophyll content than genotypes sensitive to drought [72]. In the current investigation, we found that the RWC characteristic significantly decreased under WS conditions; in contrast, an increase in the contents of chlorophyll A, B, and A + B was noted. Under WS, there was an approximate 20–22% increase in chlorophyll levels A and A + B whereas chlorophyll B increased by about 45%; [73] saw a comparable rise in maize. Under water stress conditions, the content of essential osmoprotectants, including free proline, increased in the genotypes. Plants accumulate organic osmolytes to preserve cell homeostasis, which is a well-known strategy for resisting drought stress [74]. Similar mechanisms were observed in sesame [75].

### Drought-tolerant indices

Utilizing drought-tolerant indices is crucial for developing superior cultivars that can flourish in arid environments since drought tolerance in sesame is a complex feature controlled by a variety of physiological mechanisms [72]. Studies evaluating sesame genotypes under drought stress using agro morphological features and drought tolerance indices have been few, despite the considerable genetic variety of sesame [70]. The study’s sesame genotypes’ drought tolerance indices differed significantly. Drought indicators were employed in conjunction with YP and YS to identify drought-tolerant genotypes [43]. Numerous selection indices have been proposed to choose novel genotypes based on their efficacy in both normal and drought conditions. The stress sensitivity index (SSI) and stress tolerance index (TOL) can be used to distinguish between genotypes that provide high yields under normal conditions and those that produce low yields under water stress conditions. The genotypes exhibit greater drought tolerance when SSI and TOL levels are lower [60]. Accordingly, the most drought-tolerant genotype is Shand 3. Nevertheless using just these two indices (SSI and TOL) would not effectively identify genotypes that are drought-tolerant since they will only identify genotypes that show marginal decreases in drought-related traits when compared to non-stress environments [43]. As a result, under both circumstances, these two indicators only are unable to distinguish between genotypes with high yields [76]. Many studies suggest several indices, like the geometric mean productivity (GMP), and mean productivity (MP), to solve this restriction. These indices make it possible to choose genotypes that are tolerant and have a high potential for productivity in both stressful and non-stressful environments [32, 77]. Sesame genotype H. 68 had substantially greater MP and GMP than other genotypes, with the H. 102 variety coming in second. These indicators revealed that the genotypes of H. 102 and H. 68 were more preferred and more drought-tolerant. Conversely, the genotype “N. A259” exhibits the lowest MP and GMP, making them less advantageous in these circumstances. According to some other research, the yield stability index (YSI) may be the best indicator for determining genotypic stability in both water stress and normal situations [78]. Based on our research results, “Shandweel 3” had the greatest mean YSI values. This suggests that the former genotype has the highest tolerance to water deprivation. According to [79], the Relative Drought Index (RDI) is a favorable indicator of stress tolerance. Resistance genotypes exhibited a positive DRI index [80]. This was because of their big output in both normal and stress conditions, as well as their modest averages, which is the opposite situation observed in this study. This study showed a significant and positive association between the drought indices and the yield of seeds and this is consistent with what [81] have concluded. These results offer important information for the creation of selection indices that will be utilized in the breeding of drought-tolerant sesame varieties with higher seed output and stability.

## Conclusion

To sum up, the assessment of sesame genotypes for drought resistance using agro-morphological traits, physiological traits, and drought tolerance indices has yielded important information about how sesame adapts to water deficit. Our results demonstrated a considerable variation in morphological characteristics, physiological characteristics, and drought tolerance indices across all genotypes and the water irrigation treatments under study. The highest MP, GMP, and YI indices were discovered in sesame genotype H. 68, followed by genotype H. 102. The genotype that produced the lowest SSI and TOL indices was Shandaweell 3. The average YSI readings were highest on Shandweel 3. According to our results, the genotypes utilized in this study that are most drought-tolerant are Shandaweell 3, H. 102, and H. 68. To improve breeding techniques for sustainable crop production in arid and semi-arid environments, future research should concentrate on better understanding the genetic basis of drought resistance in sesame.

## Data Availability

The data sets generated and analyzed in this study are available from the corresponding author on request.
